# Supplementary material for: Transcriptome Analysis Reveals Vimentin-Induced Disruption of Cell–Cell Associations Augments Breast Cancer Cell Migration
Source: Cells. 2022 Dec 13;11(24):4035. doi: 10.3390/cells11244035 (PMC9776984; doi:10.3390/cells11244035)
Supplement: Supplementary file 1 [file cells-11-04035-s001.zip › DEGs and enrichment analysis.zip!/DEGs and enrichment analysis/WT_VIMvsVector KEGG/ALL/WT_VIMvsVector_ALL.pathway_map.zip!/WT_VIMvsVector_ALL.pathway_map/src/hsa04514.html]

hsa04514


- hsa:4099

- Up regulated genes

ENSG00000105695(2.40379603236251)

- hsa:4099

- Up regulated genes

ENSG00000105695(2.40379603236251)

- hsa:5797

- Down regulated genes

ENSG00000173482(-3.86251572441461)

- hsa:5797

- Down regulated genes

ENSG00000173482(-3.86251572441461)

- hsa:6402

- Down regulated genes

ENSG00000188404(-2.3060730234074)

- hsa:1003

- Down regulated genes

ENSG00000179776(-4.78450423698439)

- hsa:1003

- Down regulated genes

ENSG00000179776(-4.78450423698439)

- hsa:79679

- Down regulated genes

ENSG00000134258(-3.13404991049139)

Close
